# Critics for major indexing service

**DOI:** 10.1186/s40902-021-00321-7

**Published:** 2021-09-06

**Authors:** Seong-Gon Kim

**Affiliations:** Department of Oral and Maxillofacial Surgery, College of Dentistry, Gangneung-Wonju National University, Jibyun-dong, Gangneung, Gangwondo 25457 Republic of Korea

The main task of scientific journals is publishing highly impactful articles in their field. However, publishing is not free. Financial pressure can be found from the operating submission system, article review process, editing the accepted articles, etc. The classic article has a subscription-based model, which readers pay to view. Although large companies save money by distributing labor-intensive work to the low- and middle-income countries, small regional publishers cannot endure this increase in publication fees. Accordingly, except for a few publishers, most publishers select an open-access model as their publication model. Now, only rich people can submit their work for publication to a scientific journal. At a glance, this is not fair; a big company gives a waiver for a submission from underdeveloped countries. While this is fine, the number of submissions from these countries is limited and the general quality of articles is not good enough. Thus, most editors still rely on submissions from qualified scientists, but not all of these scientists are rich. Accordingly, the article processing charge (APC) should be paid by public funds, universities, or academic societies. Otherwise, these parties will not have a chance for publication.

The article indexing service shows its power at this point. In the case of Korea, most universities support the APC only for articles published in Scientific Citation Index Expanded (SCIE) journals (operated by Clarivate Analytics). Some universities also pay for the articles indexed by Scopus (operated by Elsevier). Therefore, the editor should try their best to index their journal to one of these services. Otherwise, the number of submissions will not increase. As the importance of indexing increases, the transparency of selecting criteria is also important for maintaining the authority of the indexing services. Three years ago, the following reason for rejection was suggested by a famous indexing service: “It is a great pity that the society was advised to drop the word Korean from the title, as this gave the journal a very clear geographic distinction and discriminator from other journals, which it has now lost.” Even though we removed “Korean” from the title, I do not believe this decision damaged the quality of our journal. If this is the main reason for rejection, we will not have a second chance until correcting our journal title. This can be evidence that politics intervened in this decision process, even in major indexing services. In the case of Clarivate Analytics, the service did not communicate with the editor, who is mainly responsible for the quality control of the journal. Although *Maxillofacial Plastic and Reconstructive Surgery* (MPRS) has been indexed by the Emerging Sources Citation Index (ESCI), I have not received a single mail from Clarivate Analytics for the background of this decision. If the editor does not know the weakness of their journal, there will be no way to improve the journal.

Recently, Clarivate Analytics launched the “Journal Citation Indicator (JCI)” for evaluating both SCIE and ESCI titles. It looks like a wonderful trial and can be the first step for a fair comparison among titles. Fair competition should not be the motto exclusively for the sports game only. Some titles have extremely low impact, but still, stay in the SCIE. Without political consideration, this is poorly explained. Most major indexing services asked for a new journal to be included as the first or the second quartiles (Q1 or Q2) in terms of citations. However, it is unclear whether it should be positioned just once or should be positioned serially. There has been up and down in the citation number of indexed journals. If only new born journals should keep their status as Q1 or Q2, it should be unfair. According to JCI, MPRS has been ranked 62nd in 2019 and the 80th in 2020 among the category of the *Dentistry, Oral Surgery & Medicine*. It was the 1st-ranked journal among the ESCI indexed titles in this category in 2019 and the 6th ranked in 2020. The number of titles in this category was 163 including 13 new titles in 2020.

## Data Availability

Not applicable.

